# Behavioral evidence for inter-hemispheric cooperation during a lexical decision task: a divided visual field experiment

**DOI:** 10.3389/fnhum.2013.00316

**Published:** 2013-06-27

**Authors:** Marcela Perrone-Bertolotti, Sophie Lemonnier, Monica Baciu

**Affiliations:** ^1^INSERM U1028, CNRS UMR5292, Lyon Neuroscience Research Center, Brain Dynamics and Cognition Team, Université Claude Bernard Lyon 1Lyon, France; ^2^LUTIN Userlab - CHArt Cognitions Humaine et Artificielle, Département de Psychologie, Université Paris 8Paris, France; ^3^Laboratoire de Psychologie et Neurocognition CNRS – UMR 5105, Département de Psychologie, Université Pierre Mendès-FranceGrenoble, France; ^4^Institut Universitaire de France, IUFParis, France

**Keywords:** asymmetry, cooperation, inhibition, divided visual field, redundant, bilateral, lexical decision

## Abstract

**HIGHLIGHTS**

- The redundant bilateral visual presentation of verbal stimuli decreases asymmetry and increases the cooperation between the two hemispheres.
- The increased cooperation between the hemispheres is related to semantic information during lexical processing.
- The inter-hemispheric interaction is represented by both inhibition and cooperation.

This study explores inter-hemispheric interaction (IHI) during a lexical decision task by using a behavioral approach, the bilateral presentation of stimuli within a divided visual field experiment. Previous studies have shown that compared to unilateral presentation, the bilateral redundant (BR) presentation decreases the inter-hemispheric asymmetry and facilitates the cooperation between hemispheres. However, it is still poorly understood which type of information facilitates this cooperation. In the present study, verbal stimuli were presented unilaterally (left or right visual hemi-field successively) and bilaterally (left and right visual hemi-field simultaneously). Moreover, during the bilateral presentation of stimuli, we manipulated the relationship between target and distractors in order to specify the type of information which modulates the IHI. Thus, three types of information were manipulated: perceptual, semantic, and decisional, respectively named pre-lexical, lexical and post-lexical processing. Our results revealed left hemisphere (LH) lateralization during the lexical decision task. In terms of inter-hemisphere interaction, the perceptual and decision-making information increased the inter-hemispheric asymmetry, suggesting the inhibition of one hemisphere upon the other. In contrast, semantic information decreased the inter-hemispheric asymmetry, suggesting cooperation between the hemispheres. We discussed our results according to current models of IHI and concluded that cerebral hemispheres interact and communicate according to various excitatory and inhibitory mechanisms, all which depend on specific processes and various levels of word processing.

## Introduction

The majority of individuals show a left hemisphere (LH) predominance for language processing (Josse and Tzourio-Mazoyer, 2004). Nevertheless, both hemispheres are more or less involved during language processing and they are in constant interaction. The mechanisms underlying the inter-hemispheric interaction (IHI) is still a topic of debate (for a review, see van der Knaap and van der Ham, 2011). In the present study, and through the manipulation of the information conveyed between hemispheres by means of divided visual fields (DVF) presentation of verbal material, we evaluated both the hemispheric specialization and the inter-hemisphere interaction.

DVF is based on the anatomo-functional properties of partially crossed visual pathways (Chiarello et al., 2004; Bourne, 2006). Consequently, a briefly presented stimulus (flashed) in one's visual hemi-field is processed first by the opposite hemisphere (LH for the right visual hemi-field presentation; right hemisphere-RH for left visual hemi-field presentation). The logic behind this procedure is that visual verbal stimuli are processed faster and more efficiently if they are presented first to the specialized hemisphere to process language, generally the left one (Bourne, 2006). Studies performed with DVF procedure provide convergent evidence with those using brain lesion-deficit approach and neuroimaging studies. Indeed, DVF studies suggest that (1) LH is predominant for processing language and (2) RH has several language abilities. The degree of hemispheric specialization (LH > RH) varies according to the language task and the psycholinguistic features of the stimuli (Chiarello et al., 2005; Cousin et al., 2006; Perrone et al., 2009). These studies are suggesting a continuum of hemispheres involvement, rather than absolute unilateral hemispheric specialization (Pulvermüller, 1996; Jung-Beeman, 2005). Both hemispheres communicate continuously during language processing and show dynamic interaction (Banich, 1998). This IHI may be explored by using a specific procedure derived from DVF, the bilateral and simultaneous presentation of stimuli in both left (LVF) and right (RVF) visual hemi-fields (see Bourne, 2006 for a review). Compared to unilateral, the bilateral presentation shows higher performances for language processing. This is particularly true if bilateral presented stimuli are redundant (identical) rather than different (Banich and Karol, 1992; Hellige, 1993; Mohr et al., 2000, 2002). The gain of performance (bilateral > unilateral) is called “bilateral gain” (BG) and represents behavioral evidence for the inter-hemispheric cooperation (Zaidel and Rayman, 1994; Mohr et al., 1996; Hasbrooke and Chiarello, 1998; Weissman and Banich, 2000). Cooperation between hemispheres is based on anatomical structures connecting both hemispheres such as with the corpus callosum (Weems and Reggia, 2004; Stephan et al., 2005). Indeed, the BG is not obtained in split-brain patients (Mohr et al., 1994). To be measured, the BG requires that “hemispheres are not independent processors” (Weems and Reggia, 2004).

Nevertheless, in healthy subjects, BG was only observed under certain conditions such as the performance of complex tasks and familiar stimuli (Banich and Belger, 1990; Mohr et al., 1996). The facilitation for familiar stimuli is currently explained by the neurocognitive model based on Hebbian learning mechanisms (Pulvermüller and Mohr, 1996). This model suggests that previously learned items are stocked under memory representations of words (see Pulvermüller, 1996; Pulvermüller and Mohr, 1996; Mohr et al., 2007). These representations correspond to large networks composed of inter-connected neurons and constitute functional units distributed across hemispheres. A Functional Unit emerges from the frequent co-activation of an inter-hemispheric ensemble of neurons by the repeated presentation of stimuli. Thus, a unilateral visual hemi-field presentation may activate the corresponding functional unit distributed across hemispheres. If the same item is presented simultaneously in both visual hemi-fields, the activation of cortical representation across hemispheres could double its strength with the additive mechanisms present. In terms of performance the additive mechanisms may be reflected by the BG effect and the decrease of inter-hemispheric asymmetry (Mohr et al., 1996). In other words, the bilateral redundant (BR) presentation of stimuli increases the cooperation between hemispheres.

Even with the BG reports following the BR presentation, it remains unclear what specific type of information facilitates the inter-hemispheric cooperation during the word processing. The BR presentation involves identical perceptual, semantic and decisional processing (response making, i.e., for the two stimuli presented the same response as expected). Each type of processing may facilitate the cooperation: perceptual (see Banich and Karol, 1992) such as physical resemblance of stimuli (Fernandino et al., 2007; Baird and Burton, 2008), semantic relationship (Koivisto, 2000; Baird and Burton, 2008) and decision-making for providing responses (Banich and Karol, 1992; Iacoboni and Zaidel, 1996; Fernandino et al., 2007). In line with this proposal, Baird and Burton (2008) evaluated the nature of the inter-hemispheric cooperation during a non-verbal task. Specifically, they evaluated the effect of low sensory and high abstract level of the information transferred between hemispheres. They presented faces (familiar vs. unfamiliar) under unilateral and bilateral visual field presentation. The bilateral presentation was composed of two conditions, redundant (i.e., same face image projected simultaneously to both hemispheres) and non-redundant (NR) (i.e., different face images, each one presented to one hemisphere). In the NR condition, the two faces represented the same person (semantic similarity) taken in different positions (perceptual difference). Results revealed BG for familiar faces during both bilateral conditions presentation, redundant and NR. The authors suggest that the BG is not restricted to identical stimuli (perceptual information) but also concerns stimuli designating the same concept or same identity (semantic information). Nevertheless, although reduced, the perceptual information was still persistent during the bilateral NR presentation (i.e., same face). Consequently, the BG observed in this condition may be related to both, semantic and perceptual information shared between hemispheres.

Furthermore, Fernandino et al. (2007) investigated the IHI during a lexical decision task using a DVF experiment. Participants were asked to judge whether a target string-of-letters was a word (manual response “yes”) or a pseudo-word (manual response “no”). Target items (word or pseudo-word) were always underlined in order to be easily differentiated from the distractors. Authors used two types of bilateral presentation to evaluate the effect of “lexical redundancy” during the IHI. In one of them, the distractor had the same lexical nature as the target item (i.e., both items were lexically related; if the target was a word, the distractor is a word too, i.e., congruent distractor condition). In the other one, the distractor was lexically different from the target item (i.e., if the target was a word, the distractor was a pseudo-word, i.e., incongruent distractor condition). Thus in the bilateral congruent distractor condition, both target and distractor induced the same response decision in each hemisphere. Conversely, in the bilateral incongruent distractor condition, the target and the distractor induced a different response decision in each hemisphere. Based on this experimental configuration, the authors investigated how the lexical nature of the distractor modulates target processing by each hemisphere (visual hemi-field of presentation) at different levels (before or after response decision). Their results suggest that IHI takes place before the programming of the motor response. Specifically, they found that incongruent distractors delayed the lexical decision compared to perceptual distractors (i.e., string of XXXX in the opposite visual field).

Here we used an original paradigm based on previous studies (Banich and Karol, 1992; Fernandino et al., 2007), which manipulates the type of shared information between hemispheres during a lexical decision task (i.e., decide whether the stimulus presented is a real word or not; Chiarello, 1988). By manipulating the relationship between target and distractor, our paradigm allows us to specifically focus on perceptual characteristics (physical resemblance) of the information at pre-lexical level, on semantic characteristics (knowledge and meaning) during lexical access, and on decisional information (planning response) at the post-lexical processing. Our major aim was to determine how the type of information modulates the inter-hemispheric cooperation; we focused on the degree of hemispheric involvement, namely, the increase or the decrease of the degree of inter-hemispheric asymmetry.

## Materials and methods

### Participants

Forty native French speakers (15 males, Mean = 22.33 year, *SD* = 4.89 year) participated in the experiment. They had normal or corrected-to-normal vision and were right-handed (Mean = 98, *SD* = 15) as determined by the Edinburgh Handedness Inventory (Oldfield, 1971). All participants were undergraduate students and received course credits for their participation. They all gave informed consent to participate in the experiment.

### Stimuli

**Figure 1:**
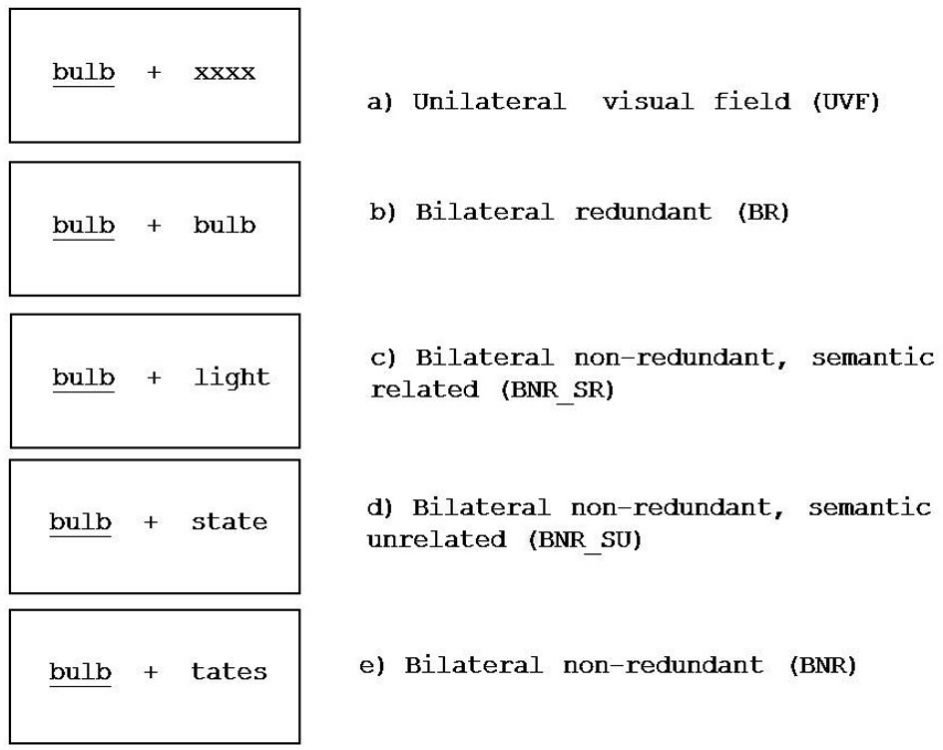
**Experimental conditions (see description in Material and Methods section)**.

Stimuli were built to meet the five modes of presentation (see Figure 1 and Experimental Conditions section). We used 128 French words and 128 pseudo-words during a lexical decision task (see Table A1). Stimuli were controlled in a number of letters^1^ (4–7), French lexical frequency (Lexique.org; New et al., 2004) and semantic relationship (Alario, 1999) according to each of the five experimental conditions (see below). The pseudo-words were built by changing three or four letters in words.

Further, 32 pairs of words were selected, from the 100 highest semantically related (SR) word pairs, from the Alario's database (Alario, 1999). The semantic association could lead to a taxonomic (apple–banana) or a contextual (apple–fruit) category. Alario's database is based on free verbal associations between names of concrete objects, made by 89 French participants. Our assumption was that the free semantic association of items favors the ecological approach of neural connectivity (neural functional units), without any prerequisite that could distort the results.

All target items were presented during the five experimental conditions. The other word of each pair was considered as distractor and presented during the bilateral semantically related (SR) condition. In addition, we selected two other words matching the distractor in terms of frequency of occurrence^2^, number of letters, lexical status and gender (see Table A1). One of these two words was presented during the bilateral semantically unrelated (SU) condition, and the other one during the bilateral NR condition. Pseudo-words were constructed from words and were presented during each experimental condition according to original words (e.g., the pseudo-word “rechai” was built from the real word “cahier,” thus “reachi” was presented in the same experimental condition presenting “cahier”). To summarize, for each target-word and according to each condition of presentation we associated 32 semantically related words (SR condition), 32 SU words (SU condition) and 32 pseudo-words (NR condition). Similarly, for each target pseudo-words we associated 64 control pseudo-words (SR and SU conditions) and 32 words (NR condition).

E-Prime software (E-Prime Psychology Software Tools Inc., Pittsburgh, USA) was used for the experimentation. Stimuli were written in black “Courier New” font size 24 and displayed on the white screen of a computer monitor (screen resolution 1024 × 768 pixels) located at a distance of 57 cm from the participant's eyes.

### Experimental conditions

We considered the following five experimental conditions according to the relationship between target (presented in one visual hemi-field) and distractor (presented in the other visual hemi-field):

1. *Unilateral left/right visual hemi-field, UVF* (Figure 1a): the target was a word or a pseudo-word and the distractor was a string of letters (letter “X” matching the number of letters in the target). The target and the distractor did not share relevant information for the task performance.
2. *Bilateral redundant, BR* (Figure 1b): the target and the distractor were presented simultaneously and were identical (same lexical category and same stimulus; i.e., the same word). The information shared between target and distractor was perceptual, semantic and decisional.
3. *Bilateral non-redundant semantically related, BNR_SR* (Figure 1c): the target and the distractor were SR words selected from Alario (1999) database. The information shared between target and distractor was semantic and decisional.
4. *Bilateral non-redundant semantically unrelated, BNR_SU* (Figure 1d): the target and the distractor had the same lexical status (i.e., if a target is a word, the distractor is a word, same lexical status but dissimilar stimuli). Word distractors were matched in terms of lexical frequency and number of letters with word distractors presented in BNR_SR condition (New et al., 2004). The information shared between target and distractor was decisional only, because both items induce the same manual response (e.g., yes).
5. *Bilateral non-redundant, BNR* (Figure 1e): the target and the distractor were lexically dissimilar (i.e., word and pseudo-word). As in the previous condition, word distractors were matched in terms of lexical frequency and number of letters with word distractors shown in BNR_SR condition. The target and the distractor did not share relevant information to perform the task.

For (4, 5) conditions, only word trials were considered for analysis, the pseudo-words were used as a control.

### Paradigm

The whole experiment was divided into four blocks, each of them including the same number of words and pseudo-words (i.e., 32 items). Furthermore, each block was performed under two modes of presentation, unilateral (UVF) and bilateral (BR or BNR_SR or BNR_SU or BNR). Indeed, each block was composed of 32 unilateral and 32 bilateral trials for one on the four bilateral conditions. Thus, there were four blocks, one for each bilateral condition. Trials were presented randomly within each block. This presentation allowed a left and right visual hemi-field presentation of each target item. The whole experiment included 256 trials.

**Figure 2:**
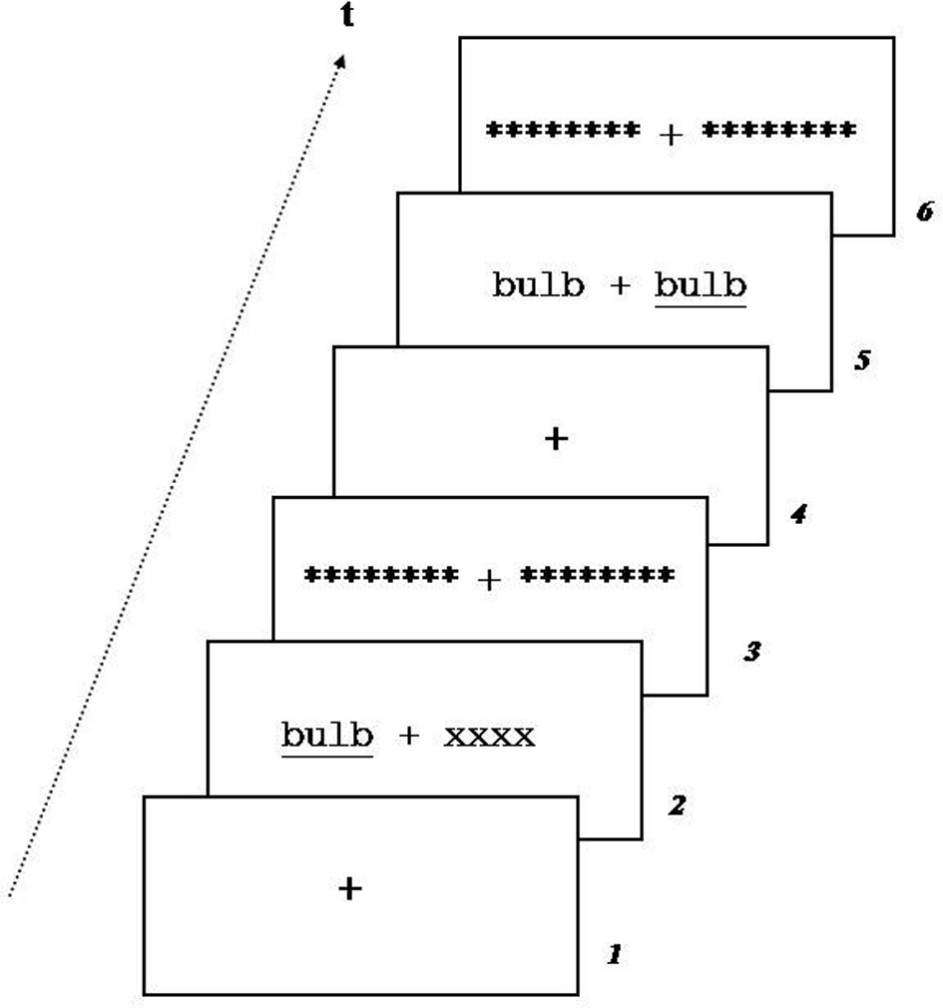
**Example of trial during unilateral (1) and (2) bilateral redundant presentation.** A fixation cross was presented for 500 ms, followed by stimulus presentation for 180 ms, mask during 30 ms and fixation cross for 1500 ms.

Each trial (Figure 2) began with a 500 ms fixation cross (in order to keep the gaze direction at the center of the screen) followed by a stimulus displayed for 180 ms, either in UVF (RVF or LVF) or simultaneously in both visual hemi-fields (RVF and LVF). The short duration of stimulus presentation insured mono-hemispheric presentation (Belger and Banich, 1992; Afraz et al., 2003). Stimulus presentation was followed by a 30 ms visual mask composed of a sequence of eight stars. The inner and the outer edges of the lateralized presented stimuli were located at 2 and 6° from the eyes fixation, respectively. The trial ended with a 1500 ms fixation cross. The target stimulus was underlined.

Participants were instructed to perform a lexical decision task based on deciding whether or not the underlined item (target) was a real French word. The task was the same for the five experimental conditions. Participants provided manual responses with their index and middle finger. The responding hand was controlled (Eviatar et al., 1997; Provins, 1997); as each participant responded with the right hand for half of the experimental blocks and switched to the left hand for each last half. Before the experiment, participants went through a short training session which included various items not shown during the experiment. The participant's reaction time (RT) and accuracy (% Correct Responses, CR) were recorded for each participant and condition.

### Data processing

A two-step ANOVAs (analysis of variance) was performed. First of all, we evaluated the degree of hemispheric specialization by considering all target items, independently of their lexical nature and for all conditions. In order to achieve this, we compared the performances for stimuli presented in the left vs. the right visual hemi-fields. Thus, if a right visual hemi-field advantage was observed which suggests LH predominance, a second level analysis was subsequently performed, this to evaluate the effect of considered variables.

Only words were considered for the second analysis. Specifically, the second level analysis was an ANOVA including all five experimental conditions according to the visual hemi-field of presentation. Four statistical contrasts were calculated according to the hypotheses: (1) BG: UVF vs. BR; (2) Effect of perceptual information: BR vs. BNR_SR; (3) Effect of semantic information: BNR_SR vs. BNR_SU; (4) Effect of decisional information: BNR_SU vs. BNR.

## Results

Accuracy (% CR) and latency (mean RT) values were included in two ANOVAs, one by participant (F1) and another one by item (F2).

### First step ANOVA: hemispheric specialization

The performances were collapsed for all conditions (unilateral and bilateral) and for all targets stimuli (words and pseudo-words). Thus, we considered visual hemi-field of presentation (RVF-LH; LVF-RH) as a within-subject factor.

#### Latency (mean RT)

**Figure 3:**
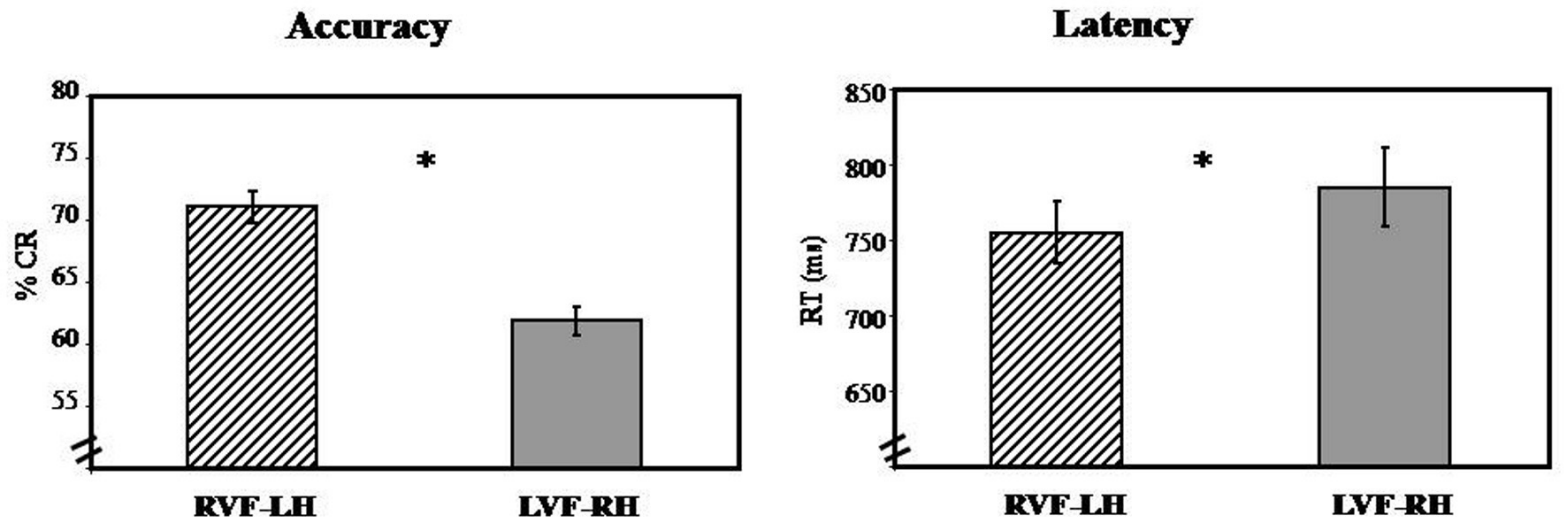
**Visual hemi-field effect: illustrates graphically results obtained in the first-step ANOVA analysis in terms of Accuracy (% CR) and response time (RT).** Abbreviations: RVF, right visual hemi-field; LVF, left visual hemi-field; LH, left hemisphere; RH, right hemisphere. ^*^p < 0.05.

In terms of RT, results reveal main effect of visual hemi-field [*F*1_(1, 39)_ = 65.24; *PRE*^3^ = 0.62; *p* < 0.05; *F*2_(1, 63)_ = 20.25; *PRE* = 0.24; *p* < 0.05] with faster responses for RVF-LH (*M* = 754.81 ms, *SD* = 20.90 ms) than for LVF-RH (*M* = 785.36 ms, *SD* = 26.12 ms), suggesting LH specialization (Figure 3).

#### Accuracy (% CR)

In terms of Accuracy, the results revealed the visual hemi-field's main effects of [*F*1_(1, 39)_ = 65.24; *PRE* = 62; *p* < 0.05; *F*2_(1, 63)_ = 32.96; *PRE* = 0.34; *p* < 0.05] with more accurate responses for RVF-LH (*M* = 71.05%, *SD* = 1.35%) than for LVF-RH (*M* = 61.85%, *SD* = 1.11%). This result suggests that lexical decision was performed more accurately when stimuli were presented first to the LH (Figure 3).

### Second step ANOVA

We considered the visual hemi-field of presentation (RVF-LH, LVF-RH) and the experimental condition (UVF, BR, BNR_SR, BNR_SU, BNR) as within-subject factors for word items. We first presented the omnibus ANOVA results according to both dependent variables. Then, we presented the statistically planned comparison according to each of our hypotheses. Significant results were only obtained in terms of accuracy. Based on latency, we did not obtain significant interaction with the omnibus ANOVA. Moreover, the planned comparisons according to each hypothesis were not significant.

#### Latency (mean RT)

In terms of RT, results revealed only a significant main effect of visual hemi-field [*F*1_(1, 39)_ = 15.71; *PRE* = 0.28; *p* < 0.05; *F*2_(1, 31)_ = 25.66; *PRE* = 0.45; *p* < 0.05] with faster responses for RVF (*M* = 728.29 ms, *SD* = 19.7 ms) than for LVF (*M* = 777.92 ms, *SD* = 24.39 ms) suggesting LH specialization. The main effect of experimental condition was not significant [*F*1_(4, 156)_ = 0.88; *PRE* = 0.022; *p* = 0.47; *F*2_(4, 124)_ = 1.74; *PRE* = 0.05; *p* = 0.14]. The interaction between the experimental condition and the visual hemi-field of presentation was not significant either [*F*1_(4, 156)_ = 0.49; *PRE* = 0.01; *p* = 0.73; *F*2_(4, 124)_ = 0.78; *PRE* = 0.02; *p* = 0.53].

#### Accuracy (% CR)

**Figure 4:**
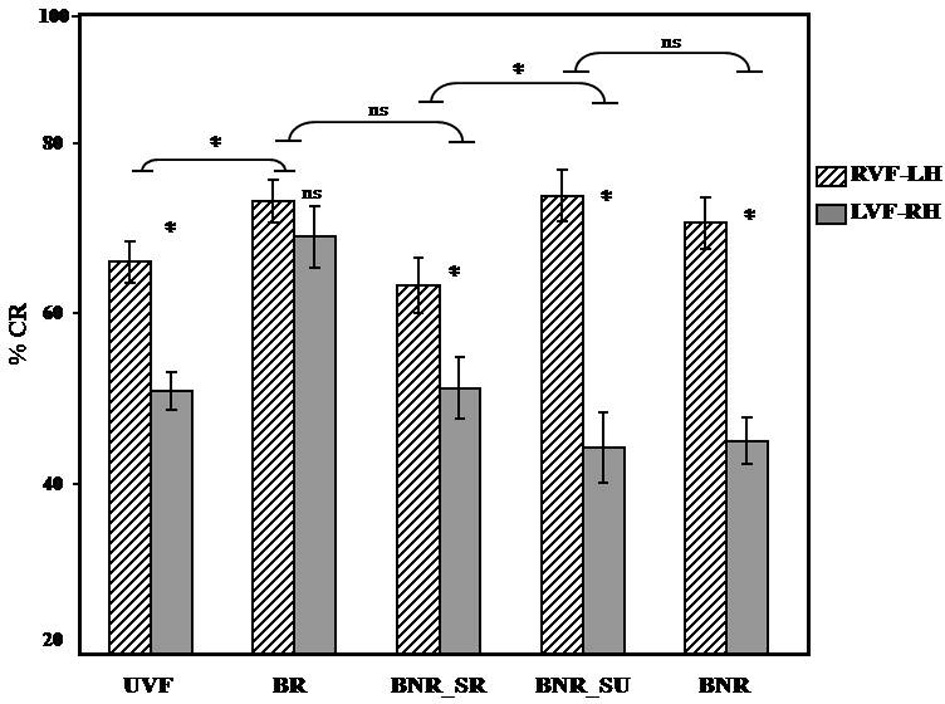
**Visual hemi-field and experimental condition interaction: illustrates in terms of accuracy the significant interaction between the visual hemi-field of presentation and the experimental condition for UVF vs. BR and BNR_SR vs. BNR_SU.** Abbreviations: UVF, unilateral visual hemi-field; BR, bilateral redundant; BNR_SR, bilateral non-redundant semantic related; BNR_SU, bilateral non-redundant semantic unrelated; BNR, bilateral non-redundant; RVF, right visual hemi-field; LVF, left visual hemi-field; LH, left hemisphere; RH, right hemisphere. ^*^p < 0.05.

In terms of Accuracy, the results revealed a significant effect of visual hemi-field's presentation [*F*1_(1, 39)_ = 49.05; *PRE* = 0.55; *p* < 0.05; *F*2_(1, 31)_ = 62.82; *PRE* = 0.66; *p* < 0.05] with more accurate responses for RVF-LH (*M* = 69.40%, *SD* = 2.09%) than for LVF-RH (*M* = 52.10%, *SD* = 2.04%). Furthermore, our results reveal a significant effect of experimental conditions [*F*1_(4, 156)_ = 10.69; *PRE* = 0.22; *p* < 0.05; *F*2_(4, 124)_ = 15.14; *PRE* = 0.33; *p* < 0.05], see Table 1. More interestingly, our results reveal a significant interaction between the experimental condition and the visual hemi-field's presentation [*F*1_(4, 156)_ = 7.66; *PRE* = 0.16; *p* < 0.05; *F*2_(4, 124)_ = 8.74; *PRE* = 0.21; *p* < 0.05], see Figure 4. Consequently, we present below (Table 1) the results for each statistical contrast, according to our hypotheses.

**Table 1:** **Summarizes the mean value and the standard (*italic values*) deviation for the dependent variables: percent of correct responses (% CR) and mean of correct response time (mean RT, ms), according to the type of presentation and the visual hemi-field/the hemisphere of presentation**.

	**RVF-LH**	**LVF-RH**	**Total**	**RVF-LH**	**LVF-RH**	**Total**
	**(%)**	**(%)**	**(%)**	**(ms)**	**(ms)**	**(ms)**
UVF	66.09	50.93	58.51	714.04	755.77	734.91
	*2.41*	*2.18*	16.23	*20.05*	*26.35*	*22.42*
BR	73.12	69.06	71.09	726.59	761.69	744.14
	*2.51*	*3.52*	19.22	*24.87*	*31.23*	*25.01*
BNR_SR	63.30	51.28	57.29	739.99	810.04	775.02
	*3.31*	*3.57*	22.89	*27.72*	*32.74*	*27.68*
BNR_SU	73.87	44.23	59.05	725.85	784.14	754.99
	*3.03*	*4.07*	26.89	*24.92*	*30.44*	*25.84*
BNR	70.62	45	57.81	734.99	777.92	756.46
	*3.05*	*2.79*	22.31	*24.88*	*32.84*	*26.90*
Total	69.40	52.10		728.29	777.91	
	*2.09*	*2.04*		*19.70*	*24.39*	

Abbreviations: UVF, unilateral visual hemi-field; BR, bilateral redundant; BNR_SR, bilateral non-redundant semantic related; BNR_SU, bilateral non-redundant semantic unrelated; BNR, bilateral non-redundant; RVF, right visual hemi-field; LVF, left visual hemi-field; LH, left hemisphere; RH, right hemisphere.

### Modulation of the IHI

#### By the type of visual presentation (UVF vs. BR)

As shown in Figure 4, we obtained significant interaction between experimental conditions (UVF, BR) and the visual hemi-field of presentation (RVF-LH, LVF-RH) [*F*1_(1, 39)_ = 4.85; *p* < 0.05; *F*2_(1, 31)_ = 6.08; *p* < 0.05] with a higher degree of inter-hemispheric asymmetry during UVF [*F*1_(1, 39)_ = 22.55; *p* < 0.05; *F*2_(1, 31)_ = 31.99; *p* < 0.05] than BR [*F*1_(1, 39)_ = 0.96; *p* = 0.33; *F*2_(1, 31)_ = 0.94; *p* = 0.33]. This result suggests supplementary recruitment of the RH with BG, reflecting increased hemispheric cooperation during bilateral presentation of linguistic stimuli.

#### By the perceptual information (BR vs. BNR_SR)

As illustrated in Figure 4 our results do not reveal a significant interaction between experimental conditions (BR, BNR_SR) and the visual hemi-field of presentation (RVF-LH, LVF-RH) [*F*1_(1, 39)_ = 1.69; *p* = 0.20; *F*2_(1, 31)_ = 2.64; *p* = 0.11]. This result suggests a lack of significant differences between both bilateral presentations. The perceptual information does not modulate the IHI.

#### By the semantic information (BNR_SR vs. BNR_SU)

As shown in Figure 4, we obtained a significant interaction between experimental conditions (BNR_SR, BNR_SU) and the visual hemi-field of presentation (RVF-LH, LVF-RH) [*F*1_(1, 39)_ = 10.69; *p* < 0.05; *F*2_(1, 31)_ = 7.99; *p* < 0.05] with a higher degree of inter-hemispheric asymmetry during BNR_SU [*F*1_(1, 39)_ = 40.09; *p* < 0.05; *F*2_(1, 31)_ = 54.96; *p* < 0.05] than BNR_SR [*F*1_(1, 39)_ = 7.87; *p* < 0.05; *F*2_(1, 31)_ = 9.18; *p* < 0.05]. This result suggests that the semantic information decreases the degree of inter-hemispheric asymmetry and modulates the IHI.

#### By the decisional information (BNR_SU vs. BNR)

As illustrated in Figure 4, our results do not reveal significant interactions between experimental conditions (BNR_SU, BNR) and the visual hemi-field of presentation (RVF-LH, LVF-RH) [*F*1_(1, 39)_ = 0.47; *p* = 0.49; *F*2_(1, 31)_ = 0.70; *p* = 0.41] suggesting that BNR_SU and BNR induce a similar degree of inter-hemispheric asymmetry. The decisional information does not modulate the IHI.

## Discussion

The aim of the present study was to explore the modulation of inter-hemispheric cooperation during a lexical decision task according to the type of visual presentation (unilateral, bilateral) and three types of information processing (perceptual, semantic and decisional). A DVF experiment was used to compare performances for unilateral vs. bilateral simultaneous presentation of stimuli.

Our results replicated previous DVF studies reporting shorter RT and increased accuracy if the target was presented within RVF-LH than LVF-RH. This is consistent with the LH advantage for language processing (Iacoboni and Zaidel, 1996; Waldie and Mosley, 2000; Barnea et al., 2005; Vigneau et al., 2011). The results showed LH predominance for both unilateral and bilateral presentations and validated the DVF experimental paradigm.

Furthermore, we obtained BG effect for the BR compared to the unilateral presentation in RVF-LH. The BG suggests inter-hemispheric asymmetry reduction and increased inter-hemispheric cooperation by a supplementary involvement of the right hemisphere (see Lindell, 2006). Indeed, no significant difference was observed between the visual hemi-fields of presentation during redundant presentation. This pattern may be explained in terms of the facilitatory mechanism of information processing during identical simultaneous stimulus presentation (Mohr et al., 1996). Further, our results suggest that the BR presentation facilitates the cooperative work of the hemispheres, which increases behavioral performances. These results are in agreement with the neurocognitive model proposed by Pulvermüller (1996) and Pulvermüller and Mohr (1996) suggesting neural additive mechanisms (Mohr et al., 1994, 1996; Pulvermüller and Mohr, 1996).

We were also interested in identifying which type of information and processes modulates the inter-hemispheric cooperation during the bilateral redundant (BR) presentation. During the BR presentation, the information addressed to both hemispheres was identical (i.e., perceptual, semantic and decisional). Indeed, several studies suggest that this information is involved at different levels of visual word recognition and involves several types of IHI (Fernandino et al., 2007; Shipp, 2011; Doron et al., 2012). Results illustrated in Figure 4 suggest that perceptual and decision-making processes are not sufficient to explain the BG observed during BR. Indeed, we did not obtain significant interaction between the BR and the bilateral non-redundant semantic related (BNR_SR) condition, suggesting that the low perceptual information at pre-lexical level cannot explain alone the BG observed during BR presentation. Thus, the information processing at pre-lexical stage does not induce inter-hemispheric cooperation (see Chiarello, 1988).

Similarly, the lack of significant interaction between NR -semantic unrelated (BNR_SU) and lexical incongruent (BNR) presentations, suggests that the decision-making requirement at the post-lexical level cannot alone explain the BG observed during BR presentation. Accordingly, the information processing at the post-lexical level during lexical decision does not induce inter-hemispheric cooperation (Weems and Zaidel, 2005).

Our results suggest that only semantic information induces a significant difference of the degree of inter-hemisphere asymmetry during BR. Indeed, SR words (BNR_SR) induce a lower degree of inter-hemispheric asymmetry than semantically unrelated words (BNR_SU). This effect may be explained by supplementary involvement of the right hemisphere known to be equipped with semantic abilities (Hutchinson et al., 2003; Graves et al., 2010; Borovsky et al., 2013). In fact, both hemispheres show semantic abilities and complementary mechanisms (Beeman and Chiarello, 1998; Chiarello, 1998; Faust and Lavidor, 2003; Jung-Beeman, 2005). The time course of semantic processing varies across hemispheres (Koivisto, 1997): it starts earlier and gains more speed in the LH than in RH (Koivisto and Laine, 1995). This observation explains not only the supplementary right hemisphere involvement during semantic processing, but also why this effect was obtained only in terms of %CR but not in terms of RT as the LH is always faster in all types of visual presentation conditions. Nevertheless, it is important to notice that the comprehension of IHI mechanisms requires that behavioral measures were coupled with electrophysiological recording (Doron et al., 2012). Indeed, inter-individual variability in terms of duration of language processing, of inter-hemispheric transfer and of response programming may constitute an important factor. All its dimensions are difficult to distinguish by using the behavioral approach solely.

These results, which reflect cooperation between hemispheres for semantic information, may be explained according to Pulvermüller and collaborators (Pulvermüller, 1996; Pulvermüller and Mohr, 1996). The relationship between words used in our study derived from a database of words SR by the free semantic association (Alario, 1999). The semantic relationship between two words may activate a specific cortical representation (functional unit) since this semantic association is frequently used (Borovsky et al., 2013). Consequently, strong connections between cortical representations of word-pairs may be created and reinforced based on a large number of multimodal associations (Pulvermüller, 2012).

Interestingly, for unrelated semantic words (BNR_SU) and also for lexical incongruent stimuli (BNR) presentations, increased RVF-LH performances were observed. Both BNR_SU and BNR condition revealed greater inter-hemispheric asymmetry and suggested that the enhancement of the LH predominance may be detrimental for the right hemisphere performance. Surprisingly the LVF-RH performance was above the chance level (<50%) during these conditions (BNR, BNR_SU) compared to other experimental conditions (Table 1). This pattern of hemispheric involvement may reveal that two different words (lexically congruent) or two lexically dissimilar items may induce IHI. However, this interaction is mainly inhibitory (see for a review Bloom and Hynd, 2005). Indeed, Fernandino et al. (2007) established that only lexical congruent and incongruent distractors would slow down the target processing, but not the perceptual distractors. According to hemispheric independence model (Iacoboni and Zaidel, 1996; Fernandino et al., 2007; see also Weems and Reggia, 2004), the performance of a lateralized task induces inhibition of the contralateral non-predominant hemisphere in order to reduce the interference and to increase the performance. Indeed, it is possible that LH starts to process the incoming information early, leading to the inhibition of other incoming information from the RH.

## Conclusion

This study explored the modulation of IHI during a lexical decision task by using a DVF procedure with BR presentation of stimuli. Our results confirmed two well-documented results: (1) LH lateralization for a lexical decision and (2) increase of hemisphere cooperation BG during BR presentation(s). Specifically, we investigated the effects of the type of information (perceptual, semantic and decisional) on the BG. Our results suggested that perceptual and decision-making information were not sufficient to explain the IH cooperation. These show the IH cooperation is less likely to emerge during pre-lexical (perceptual) and/or post-lexical (decision-making) processing but were so, mainly during lexical semantic processing, when the semantic information was shared between hemispheres. During the lexical processing, we can explain these results in terms of facilitatory mechanisms of cooperation and supplementary right hemisphere recruitment. Overall, our results indicated that the interaction between hemispheres may follow various mechanisms, some inhibitory and others facilitatory. Additional experiments will be needed to increase the robustness of these results.

### Conflict of interest statement

The authors declare that the research was conducted in the absence of any commercial or financial relationships that could be construed as a potential conflict of interest.

## Footnotes

^1^To ensure that our results are not imputable to the number of letters (NL) composing the items, a complementary analysis has been done. Specifically, we performed an ANOVA by item in terms of accuracy (% CR) and latency (RT ms). ANOVA (Analysis of variance) included the NL (4–7) and the visual field of presentation (left, right). The results indicated lack of main effect of the NL (in terms of % CR, *F*2_(3, 60)_ = 1.82, *p* = 0.15 and in terms of RT, *F*2_(3, 60)_ = 1.64, *p* = 0.18). Also, the NL does not interact with the visual field of presentation neither in terms of % CR, *F*2_(3, 60)_ = 0.88, *p* = 0.45 nor in terms of RT, *F*2_(3, 60)_ = 1.73, *p* = 0.17).

^2^We also verified that distractors were not significant different in terms of lexical frequency *F*2_(2, 62)_ = 0.11, *p* =0.89.

^3^Instead of presenting Eta squared (used to illustrate the size effect) we present the percentage of reduction of error (PRE). Indeed, Greek letters (e.g., Eta squared) correspond to the mathematical formalization for population values (that we cannot know) and not the values for the sample. For this reason, we used the PRE that corresponds to the sample value of the partial Eta squared (Judd and McClelland, 1989, see Muller and Butera, 2007, Note 5).

## Appendix

**Table A1:** **Summarizes stimuli used in the five conditions according to lexicality (words *vs.* pseudo-words)**.

**Frequency of semantic association (%)**	**Target-word**	**Lexical frequency**	**SR distractors**	**Lexical freq.**	**SU distractors**	**Lexical freq.**	**NR (non-word) distractors**	**Target-non-word**	**SR distractors**	**SU distractors**	**NR (words) distractors**	**Lexical frequency**
36.0	cahier	31.55	école	143.99	armée	146.55	namma	rechai	elocé	eréma	maman	144.39
	homework		school		army						mother	
	book											
39.3	ancre	5.81	Bateau	82.36	animal	82.64	ideura	narce	auteba	milana	rideau	84.53
	anchor		boat		animal						curtain	
39.3	canon	48.99	guerre	354.59	raison	308.78	cohube	nacon	reruge	osiran	bouche	283.45
	gun		war		reason						mouth	
39.3	disque	42.16	musique	114.32	chaleur	116.22	sedenco	quisde	quisemu	relachu	seconde	121.49
	disc		music		heat						second	
39.3	rateau	2.50	pelle	15.61	magie	15.20	eripo	ateura	leple	egami	poire	15.07
	rake		shovel		conjuring						pear	
41.5	coffre	29.32	fort	212.77	près	285.41	sipu	ceffro	roft	spèr	puis	272.77
	box		safe		near						then	
41.5	collier	20.00	perle	23.85	roche	23.38	épeta	rilloce	lerpe	horec	étape	23.65
	necklace (string of)		pearls		rock						stap	
41.5	moulin	19.05	vent	220.27	état	218.18	ness	niloum	tnev	atét	sens	718.78
	mill		wind		state						way	
42.7	vache	53.45	lait	62.91	banc	66.42	gnar	cheva	tila	canb	rang	58.24
	cow		milk		bench						row	
42.7	clou	27.50	marteau	15.61	palmier	14.26	noyetci	luco	ateurma	malirpe	citoyen	14.73
	nail		hammer		palm						citizen	
43.8	toupie	3.92	tourner	377.09	écouter	314.05	quetrit	epouti	rountre	rétouce	quitter	318.72
	top		to turn		to listen						to leave	
					to							
43.8	valise	70.34	voyage	140.07	ventre	141.96	fetorf	sileva	gavoye	trenve	effort	136.62
	suitcase		trip		stomach						effort	
45.0	poignée	46.08	porte	617.43	fille	592.23	retre	génoipe	trope	lifle	terre	452.91
	handle		door		girl						ground	
46.1	balle	94.59	tennis	13.24	violon	13.31	osinpo	lebla	sinten	loivon	poison	12.23
	ball		tennis		violin						poison	
47.2	bureau	150.07	travail	263.45	nouveau	185.61	slipari	ureuba	lavatri	ovauneu	plaisir	233.85
	office		work		new						pleasure	
48.3	chien	184.59	chat	130.74	rêve	128.58	armi	nechi	thac	érve	mari	124.26
	dog		cat		dream						husband	
48.3	rose	57.30	fleur	164.39	ligne	162.30	serou	erso	rufle	gline	sœur	161.01
	rose		flower		line						sister	
48.3	pneu	17.03	voiture	283.11	fenêtre	280.20	imesane	neup	touvire	êtrenfe	semaine	197.50
	tyre		car		window						week	
49.4	tente	26.22	camping	3.24	concept	3.24	livacer	teten	gnapimc	poccent	clavier	3.58
	tent		camping		concept						keyboard	
49.4	tomate	13.31	rouge	68.38	riche	65.47	ugave	amotte	guero	hirce	vague	73.58
	tomato		red		rich						wave	
55.0	reveil	28.58	matin	396.76	amour	403.92	reuco	vilere	tinam	raumo	cœur	400.74
	clock		morning		love						hear	
56.1	sapin	26.28	noël	6.69	jury	5.27	ledu	pisan	ëlon	rujy	duel	5.95
	fir tree		Christmas		jury						duel	
57.3	montre	47.84	heure	924.05	femme	995.74	hosec	emtron	eruhe	emfme	chose	1057.64
	watch		hour		woman						thing	
59.5	ampoule	19.12	lumière	274.26	famille	274.39	afifera	loupame	èrlimeu	limalfe	affaire	253.38
	bulb		light		family						business	
60.7	Timbre	20.81	poste	90.68	glace	91.01	gaple	bemtir	pesto	cagle	plage	86.89
	postmark		post		ice						beach	
68.5	berceau	13.31	bébé	45.20	écho	45.95	tlof	recaube	ébbe	cého	flot	45.95
	cradle		baby		echo						flood	
69.7	laitue	2.43	salade	24.26	flaque	23.99	hocude	etailu	dalesa	quafle	douche	23.85
	lettuce		salad		puddle						shower	
70.7	serrure	19.26	clef	44.86	base	44.46	adet	rureser	flec	seba	date	45.74
	keyhole		key		base						date	
73.0	ciseaux	13.85	couper	140.88	plaire	139.80	verori	xaceusi	peroc	ralipe	revoir	36.01
	scissors		to cut		to like						to see again	
74.1	luge	1.55	neige	80.88	honte	83.45	sasem	gule	egine	theno	masse	79.80
	sled		snow		shame						mass	
82.0	ruche	3.51	abeille	9.86	clameur	9.73	birunet	cehur	lebelai	lurcame	tribune	8.99
	beehive		bee		clamor						stand	
92.1	album	18.38	photo	94.59	lampe	93.11	efuta	lambu	hopto	maple	faute	95.20
	album		photo		lamp						error	

Abbreviations: Freq., lexical frequency (Lexique.org; New et al., 2004). BNR, bilateral non redundant; SR, semantic related; SU, semantic unrelated. Semantic association: weight of semantic relationship between the target and the distractor in BNR_SR condition (Alario, 1999).
